# Inferring the role of pit membranes in solute transport from solute exclusion studies in living conifer stems

**DOI:** 10.1093/jxb/eraa058

**Published:** 2020-02-05

**Authors:** Dongmei Yang, Kailu Wei, Junhui Li, Guoquan Peng, Melvin T Tyree

**Affiliations:** 1 College of Chemistry and Life Sciences, Zhejiang Normal University, Jinhua 321004, China; 2 University of Essex, UK

**Keywords:** Centrifugation techniques, functional role of pits, lignified walls, *Metasequoia glyptostroboides*, solute exclusion, solute-free space

## Abstract

The functional role of pits between living and dead cells has been inferred from anatomical studies but amassing physiological evidence has been challenging. Centrifugation methods were used to strip water from xylem conduits, permitting a more quantitative gravimetric determination of the water and solid contents of cell walls than is possible by more traditional methods. Quantitative anatomical evidence was used to evaluate the water volume in xylem conduits and the water content of living cells. Quantitative perfusion of stems with polyethylene glycol of different molecular weight was used to determine the solute-free space. We measured the portioning of water and solute-free space among anatomical components in stems and demonstrated that lignin impeded the free movement of solutes with molecular weight >300. Hence, movement of large solutes from living cells to xylem conduits is necessarily confined to pit structures that permit transmembrane solute transport via primary walls without lignin. The functional role of pits was additionally indicated by combining data in this paper with previous studies of unusual osmotic relationships in woody species that lack pits between dead wood fibers and vessels. The absence of pits, combined with the evidence of exclusion of solutes of molecular weight >300, explains the unexpected osmotic properties.

## Teaser

Lignified cell walls appear to be impermeable to solutes with molecular weight >300, so ray cell to xylem vessel transport is restricted to unlignified pit membranes.

## Introduction

Relatively little quantitative information is available for the role of plant cell walls in water and solute transport in plants, except the obvious consideration that if water and solutes pass through a plasma membrane, then the water and solutes must also pass through the cell wall immediately adjacent to the membrane. Cell walls are typically 0.5–1.5 µm thick in living leaf cells (John *et al.*, 2013). The cell walls in leaves provide structural support and control the water relations of cells through turgor pressure. When exposed to water, the leaf cells swell in volume, stretching the cell wall, and the elastic stretch transmits a restoring pressure force or turgor pressure to the osmotic fluids in the walls. Thus, cell walls play a vital role in the water balance of leaf and root cells. However, in woody stems, the walls tend to be much thicker and more heavily lignified because the stem cell walls provide the additional functional role of mechanical support for shoots. Large leaf or shoot masses cause compressive loads on large stems, and plants taller than approximately 0.7 m cannot be supported against Earth’s gravity without lignified wood (Niklas, 1992).

**Fig. 1.**
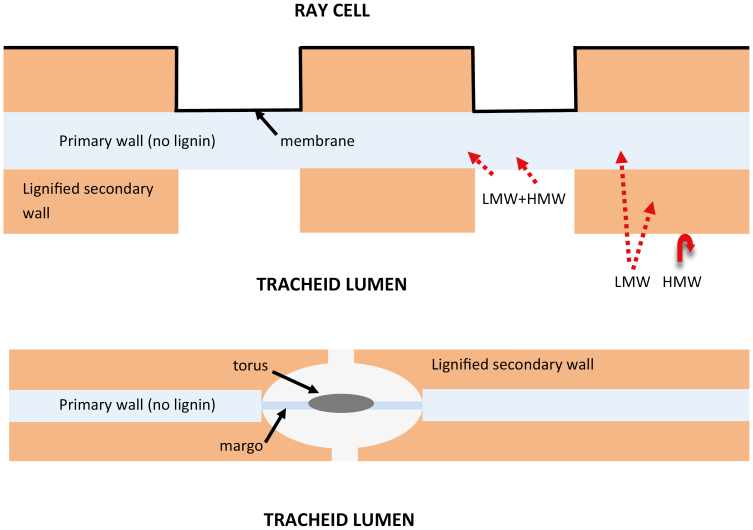
Simplified diagram of simple pits between ray cells and tracheids, and of bordered pits between adjacent tracheids. The black line labeled ‘membrane’ refers to the plasma membrane in the ray cell. Dotted arrows indicate proposed pathways of low molecular weight (LMW) and high molecular weight (HMW) solutes into the non-lignified regions and of LMW solutes into the lignified regions, and the curved arrow indicates exclusion of high molecular weight (HMW) solutes from the lignified regions. Plasmodesmata between adjacent living cells are not illustrated above because symplastic transport between living cells is not the subject of this paper, but is of physiological importance for transport from phloem to ray cells deep in the wood.

Cell walls in both stems and leaves tend to have copious pits between cells. The primary cell walls in pits, which are produced during cell division and volume growth, tend to be thinner and free of lignin; the lignified wall is laid down after cell division and volume growth. The ‘woody’ wall between two adjacent cells is a sandwich of two lignified layers with a layer of unlignified wall in the center. Pits, which cut through the lignified layers, give the visual impression that they must provide the preferred and hence the more efficient pathway for water and solute transport between adjacent cells. Lignified walls are presumed to be less permeable to solutes than primary cell walls of pits; however, quantitative experimental verification is challenging. One proof would be to demonstrate exclusion of large solutes from lignified secondary walls, and that is the purpose of this study. Figure 1 is a schematic diagram of pits together with the hypothesis of the permeability of pits versus lignified walls. Cell walls are generally thought to be more permeable to solute and water transport than plasma membranes by at least an order of magnitude for water and more orders of magnitude for solutes, yet pits are present even between dead cells (tracheids or vessel lumina) and adjacent living cells (ray cells and xylem parenchyma). There must be something about lignified cell walls that make them particularly impermeable to higher molecular weight (MW) solutes but still permeable to water and small solutes. The higher MW substances are thought to move between adjacent living cells through plasmodesmata via the symplast, which allows them to avoid penetration through cellulose walls and plasma membranes, but functional plasmodesmata do not occur between living cells and dead xylem cells (dead tracheids and vessels) even though the dead cells are physiologically functional. Pit membranes often have ‘protective layers’ that are composed of pectins and are thought to play a role in formation of tyloses in angiosperms; this layer is not shown here and is unlikely to occur in the gymnosperm species used in this study.

Solute-free space in the context of cell walls is defined as water spaces large enough to hold small solutes but not large ones. If lignified cell walls are also impermeable to large solutes, then one should be able to demonstrate this via quantitative perfusions of stems with low- versus high-MW solutions. Cell walls contain a high concentration of weak-acid polymers, which form the salts of acids at solution pH values >5.5, and the concentration of the weak-acid groups is 0.3–1 molal (Grigon and Sentenac, 1991; Meychik and Yermakov, 2001). Hence, cell walls act as weak cation exchange sites that repel anions and concentrate cations. Hence, salts and weak acids are not good candidates for measuring the volume of ‘solute-free space’ in cell-wall water.

We used polyethylene glycol (PEG) as a quantitative probe of the solute-free space because PEG is a xenobiotic unlikely to be accumulated or metabolized by living cells, is available in a variety of useful MWs (from 200 to thousands), is highly soluble in water, and is uncharged. A few new and interesting insights are gained about the role of pits and cell walls in the transport of solutes in and out of ray cells in tree stems. The insights follow from the comparison of PEG experiments to previously published experiments from Tyree’s group concerning the unusual physiology (i.e. stem pressure in *Acer* species) of trees induced by the absence of pits at strategic locations (Cirelli *et al.*, 2008).

While studies of wet-digested or of oven-dried heartwood in the modern literature give some interesting insights into the nano-structure of dead heartwood or delignified pulp wood, little of this literature is relevant to the biological behavior of living sapwood, which is the focus of this study. However, the discussion will briefly introduce some of the wood technology literature that may have techniques that could be adapted for future studies of wood containing living cells.

## Materials and methods

The approach to measuring solute-free space requires four steps: (i) the quantitative microscopic measurement of lumina and cell walls of woody stems including ray cells, pith cells, and tracheids; (ii) a centrifugation technique to remove lumen water, followed by gravimetric measurements to determine cell-wall water via the initial weight of the centrifuged stems minus the dry weight after oven drying; (iii) the quantitative measurement of the maximum uptake of PEG in stems, which requires data for the influx and efflux flow rates of PEG and concentrations; and (iv) the use of kinetic models of the process that interpret the PEG mass increase in stems based on the dynamics of bulk fluid flow through tracheid lumina and diffusion of PEG into cell-wall water spaces.

### Plant materials and sampling

This study was conducted from October of 2016 to September of 2017 on the campus of Northwest A&F University, in Yangling, Shaanxi, China (34° 16′ N, 108° 4′ E). The study focused on the conifer species *Metasequoia glyptostroboides* Hu and W. C. Cheng after a survey of many other species to eliminate those with resin and too high a fraction of living ray and pith cell tissue within the stem. Stem samples approximately 90–120 cm long with a 7–9 mm basal diameter were collected from sun-exposed branches in a canopy of large mature trees in the early morning when water potential was at its maximum. Shoots were sprayed with water, then enclosed in black plastic bags with wet paper, and transported to the laboratory within 15 min. The cut ends of branches were recut under water and placed in water to release the tension; then, the side branches and leaves were excised using razor blades. Stem segments 27.35 ± 0.05 cm long and 6–7.5 mm in diameter were cut under water for later centrifugation and PEG accumulation measurement. All lab measurements were made at 25 °C to avoid temperature effects on measurements of flow-rate, which changes hydraulic conductivity by >2% per °C.

### Inducing cavitation in tracheid lumina to assess cell-wall water content

A centrifugation method was used to drain tracheid lumina of water via tension-induced cavitation and the embolism process (Cochard, 2002; Cochard *et al.*, 2005). The lumen-drained samples could then be used to assess residual water content from fresh weight minus dry weight measurements. The principle of the method is to spin a stem segment in a custom-designed centrifuge rotor (ChinaTron, Cense Inc., Changsha, China), as described in Wang *et al.* (2014). While spinning, the tension induced by the increased revolutions per minute causes a measurable loss of hydraulic conductivity using the method described in Wang *et al.* (2014). When the percentage loss of hydraulic conductivity reached >99%, the tracheid lumina were assumed to be drained of all water near the axis of rotation (Cai and Tyree, 2010).

Cavitation induction was conducted on 2- or 3-year-old stems because 1-year-old shoots were normally too short and narrow. Before the measurement started, the stems were flushed with 0.01 M KCl (prepared with ultrapure water) at an applied pressure of 0.2 MPa for 30 min. All stem samples were embolized until the percentage loss of water conductivity (PLC) exceeded 99%. Then, the shoot was removed from the centrifuge, the central 3 cm long segment was excised centering on the axis of rotation, and the bark was removed. The de-barked segment was weighed to obtain an initial fresh weight (FW) on an analytical balance to ±0.1 mg. The length and diameter of the segment were measured with a caliper (accurate to 0.1 mm) to obtain the volume *V*_wood_. Finally, the dry weight (DW) of the stem segment was determined by drying it at 75 °C for 48 h. The difference between FW and DW represented the water content in the cell walls plus the residual water in the lumina of the living ray cell and pith cell.

### Anatomical measurement of wood volume components

The wood contains the following volumetric components: tracheid lumina, total cell wall (cell-wall solids, cell-wall liquids), ray-cell lumina, and pith lumina. The primary purpose of the anatomical measurements was to provide anatomical volumetric data that could be combined with gravimetric data (FW and DW) to calculate the volume of solids and water in the cell-wall fraction. Anatomical measurements were made on the 3 cm-long central segments harvested from the centrifuge and weighed as explained above before further dehydration. A microtome (Leica RM2235, Nussloch, Germany) was used to cut 12 μm-thick sections from the selected segments. Sections were stained with 0.02% (w/v) basic fuchsin dying solution for a few seconds; then, the surface excess stain was removed by rinsing in water, and sections were washed in graded ethanol (35%, 50%, 75%, and 95%) and mounted in glycerin on glass slides. Sections were photographed under a microscope (Zeiss, Imager A.2 Göttingen, Germany) with a digital camera (Infinity 1-5C, Regent Instruments Inc., Quebec, Canada) at ×50 magnification for the whole section and at ×200 magnification for tracheid lumen and ray-cell lumen measurements. Six cross sections derived from six stem samples were prepared, and for every section, 10 regions per section with a uniform distribution of tracheids and ray cells were selected. Then, the tracheid and ray cell lumina (excluding the cell wall), pith area, and whole wood area were measured with WinCELL software (Regent Instruments Inc., Quebec City, Canada) and Image-Pro Plus 6.0. Contributions of different components of cells within the whole wood section were calculated as the percentages of different types of cell area to the total wood area. The tracheid lumen volume fraction (VF_tr_) was estimated as tracheid lumen area/wood area. This calculation ignored tracheid end-wall thicknesses, which were <0.2% of tracheid lengths. Similarly, ray-cell volume fraction (VF_r_) is ray-cell area/wood area; pith volume fraction (VF_p_) is pith area/wood area; and cell-wall volume fraction (VF_cw_) is 1−VF_tr_−VF_r_−VF_p_.

In *Metasequoia*, the ray-cell lumen is not clearly visible in cross section, so the longitudinal (radial) sections were used for ray-cell lumen area measurements because we found in other species, such as gingko, that the ray-cell fraction was not significantly different when measured in cross section versus longitudinal (radial) section.

### Cell-wall water content per wood sample volume

After the cavitation-inducing process, the tracheid lumen was drained of all water, as deduced from >99% PLC, but the centrifugal tension was insufficient to drain cell-wall water. For the pith cell, after spinning to >99% PLC, the anatomy of the pith at the axis of rotation was investigated using a stereomicroscope (Zeiss, Stemi 2000, Germany) at ×40 magnification and compared with the end segments where there was no tension; the purpose was to check if the pith cell water in the center of the stem could be drained during the experiment.

### The measurement of solution concentration using a Brix refractometer

A Brix ‘gauge’ is a handheld refractometer calibrated to measure sugar concentration. For sugar, 10 Brix=10 g sugar/(10 g sugar+90 g water)=10% concentration (solute mass/100 g solution). However, for different MWs of PEG, the masses of solutes and waters added have to be adjusted compared with sugar to yield a refractive index of 10 Brix (*C*_f_). The measured PEG concentrations equal to 10 Brix are given in Table 1. Three MWs of PEG were used: 200, 800, and 1540, named PEG200, PEG800, and PEG1540, respectively. The age and diameter of samples used were the same as used in the centrifuge water extraction experiments (5.5–7.5 mm, 2–3 years old, and 27.4 cm long).

**Table 1.** Measured solution properties for solutions having a refractive index of 10 Brix

PEG	*C*_f_	g solute	g H_2_O	% PEG	Density (g ml^−1^)
200	1.22807	21	150	12.28%	1.0151
800	1.12734	18.06	142.14	11.27%	1.0143
1540	1.12734	18.06	142.14	11.27%	1.0137

PEG: molecular weight; % PEG: g PEG per 100 g of solution×100. Densities were measured gravimetrically using a 50 ml volumetric flask after correcting for the buoyancy of 50 ml of air (0.0584 g per 50 ml). C_f_: concentration factor, the ratio of concentrations of PEG to sugar that has a refractive index of 10 Brix.

### PEG accumulation in cell walls using mass balance calculations

A mass balance approach was used to compute PEG accumulation. The mass inflow rate was measured every minute from a container of 10 Brix PEG on a balance. Let *C*_i_ be the inflow concentration (g PEG/g solution) from Table 1, and *F*_i_ (g min^−1^) be the mass of solution that enters the sample per minute; then, the inflow mass of PEG in time Δ*t*=Δ*tF*_i_*C*_i_. The outflow rate could not be measured simultaneously with the outflow concentration, *C*_o_, in Brix. Conservation of volume was presumed because evaporation of water through bark was minimal and excised side branches were sealed. The values of *C*_i_ and *C*_o_ were a linear function of the solution’s Brix value, *C*_i_ or *C*_o_=*C*_f_ Brix (g PEG/100 of PEG solution), where *C*_f_ is from Table 1. The mass balance used to compute the accumulation of PEG in the stem is given by:

$$\begin{array}{l} M_{PEG}=\sum \Delta t(F_{i}C_{i}-F_{o}C_{o})/100\;\;\;≅\sum \Delta tF_{i}(C_{i}-C_{o})/100 \end{array}$$ (1)

The division by 100 yields concentration in g PEG per g of solution. The second term in Eq. (1) is an approximation good to about 1% error because we assume that the flow rate of solution (g min^−1^) is equal for inflow and outflow (*F*_i_=*F*_o_). The biggest error is for PEG200, where the density of the outflowing solution ranges from approximately 1.00 (pure water) to 1.015 for 10 Brix PEG. We can correct for density changes by assuming an approximate conservation of volume flow in and out. Then, we have *F*_o_/ρ _o_=*F*_i_/ρ _i_ or *F*_o_=*F*_i_ρ _o_/ρ _i_ if the density of PEG is a linear function of the Brix concentration from 1 g ml^−1^ at zero Brix to 1.015 g ml^−1^ at 10 Brix (Table 1). However, our calculations show this measurement result has a negligible impact on the value of *M*_PEG_ calculated from Eq. (1).

An analytical balance with a resolution of ±0.1 mg was used to measure the influx flow rate of the PEG solution passing through the sample. The 10 Brix PEG solution reservoir was put on the balance at approximately 1 m height and flowed through the sample, which provided a pressure-head for continuous flow of solution. The concentration of PEG at the efflux end was measured with a traditional handheld refractometer every minute in the first 2 h when the concentration was changing quickly; then it was recorded every 2–3 min. Measurements continued until the efflux solution concentration was 10 Brix and equaled the influx solution concentration. The downstream end of the flow path should be the last to equilibrate PEG between the tracheid lumina and cell walls. To confirm equilibration, in some experiments flow was stopped for 30–60 min after the outflow Brix value equaled 10. Then, the flow was resumed briefly to collect a few drops of solution for Brix determination; the value never changed, which was proof of equilibrium. After the accumulation experiment, the wood sample volume was measured without bark using the water displacement method. The whole experiment was carried out at 25 °C in a laboratory. The height of the 10 Brix PEG solution reservoir was determined based on the pressure (calculated by the ratio of the flow rate to maximum hydraulic conductivity). The target flow rate was equal to approximately three times the tracheids’ lumen volume per hour in the 27.3 cm stem segments.

### Wood volume measurement

The wood volume (without bark) was measured by the water displacement method to ±1 mg. The initial weight, *W*_i_, of a 250 ml measuring cylinder was recorded filled with water to within 2 cm of the top. Then, the wood sample was lowered into the water with a small syringe needle affixed to the distal end. The final weight, *W*_f_, was measured while the wood was immersed to the level of the needle. The wood volume was calculated by *V*_wood_=(*W*_f_*−W*_i_)/0.9971, where the divisor was the water density at 25 °C.

### Data analysis

Student’s *t*-test was used for significance tests of the data.

## Results

### Impact of spinning on the *Metasequoia* pith

**Fig. 2.**
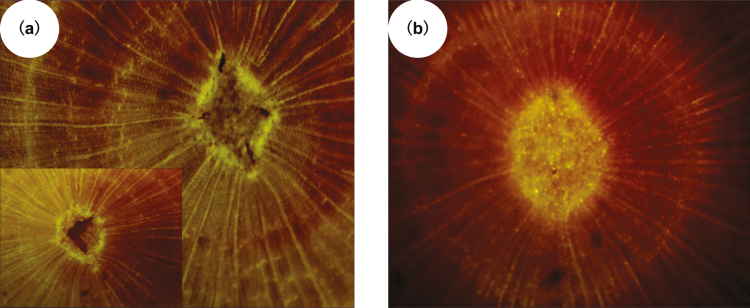
*Metasequoia* pith sections after spinning. (A) At the axis of rotation of two stems. (B) The stem ends that were immersed in water in a cuvette during spinning to 99% percentage loss of water conductivity, corresponding to a tension of up to approximately 4.3 MPa.

After spinning several replicate stem segments to >99% water conductivity loss, the central 3 cm segment had pith tissue shrunken compared with the ends. Surface photographs of the pith region are shown in Fig. 2. The pith at the axis of rotation, where the tension is highest, was damaged by the centrifugal force of the spin (Fig. 2A); however, the sample ends, which experienced no tension, had normal-looking pith such as that in freshly harvested and flushed stems (Fig. 2B). The shrinking near the axis of rotation was caused by the tension-induced negative xylem pressure of −4.3±0.2 MPa, which caused centrifugal desiccation of pith cells. The results demonstrated that substantial water in the pith of the stem’s center was extracted during spinning, although the exact quantity of the water loss could not be determined from the images.

### Quantitative anatomical observations on tracheid lumina, ray cell, pith, and cell wall

Quantitative anatomical analysis revealed that the sequence of volume fractions is ray cell<pith<tracheids lumen<cell wall (Table 2). Table 2 also shows the gravimetric determination of cell-wall water content per unit of wood volume obtained following centrifugation to extract >99% of the tracheid lumen water (*W*_cw_, %). This gravimetric determination included a small amount of water in the lumina of the ray and pith cells. The ray cells were tightly packed with starch grains. The pith cells were less packed with starch grains but were ruptured during centrifugation (Fig. 2) and presumably had little water in the 3-cm samples of stem segments excised from the axis of rotation. Assuming almost all the water determined gravimetrically in *W*_cw_ was in the anatomically determined volume fraction of the cell walls (*VF*_cw_), the cell-wall water was 39% of the cell-wall volume in *Metasequoia*.

**Table 2.** Anatomical values compared with gravimetric values

Traits	Mean	SE	*n*
*D*_tr_ (μm)	13.48	0.36	131 183
*D*_r_ (μm)	9.86	0.32	3106
VF_r_ (%)	1.76	0.15	6
VF_p_ (%)	3.61	0.91	6
VF_tr_ (%)	33.72	0.81	6
VF_cw_ (%)	60.91	0.75	6
*W*_cw_ (%)	23.89	0.82	6
*W*_cw_ */*VF_cw_ (%)	39.2	1.4	6
*N*_tr_/mm^2^	2464.46	44.33	60
δ _tr_ (μm)	3.53	0.030	1455

*D*_tr_, average tracheid lumen diameter; *D*_r_, average ray-cell diameter (the diameters were computed from the cross-sectional areas and converted to equivalent circle diameters); VF_r_, ray-cell volume fraction of the wood volume; VF_p_, pith volume fraction of the wood volume; VF_tr_, tracheid lumen volume fraction of the wood volume; VF_cw_, cell-wall volume fraction of the wood volume; *W*_cw_, cell-wall water weight after centrifugation, (FW−DW)/tissue volume (g H_2_O cm^−3^ wood) expressed as % of wood volume; *W*_cw_/VF_cw_, cell-wall water weight per cell wall volume=((FW−DW)/tissue volume (g H_2_O cm^−3^ wood))/VF_cw_, expressed as % of cell wall volume; *N*_tr_/mm^2^, number of tracheid per mm^2^; δ _tr_, tracheid wall thickness/2, or half-wall thickness; *n*, the number of observations, i.e. number of values measured in the ‘traits’ column.

**Fig. 3.**
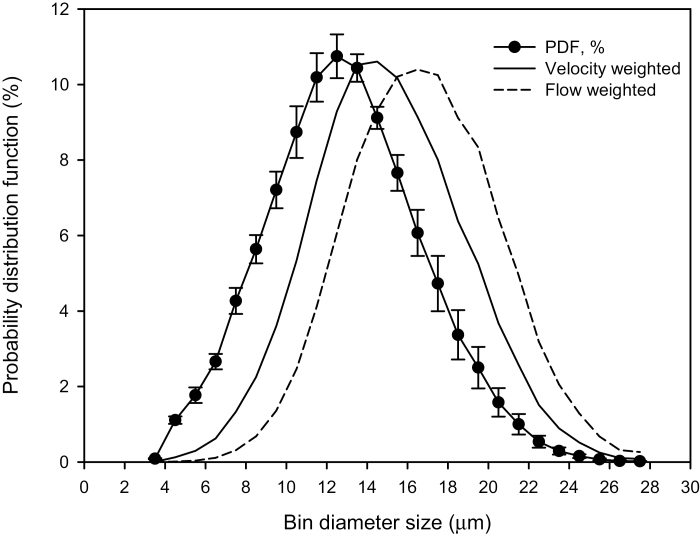
Tracheid lumen diameters were divided into 25 bin diameter size classes of 1 μm width (*x*-axis), and the percentage of lumina in each diameter size class was computed (*y*-axis). The average sap velocity and the sap volume flow rate are also shown as PDF values. The velocity was proportional to diameter squared (*D*^2^), and the flow rate was proportional to *D*^4^, as follows from Poiseuille’s law. These PDF functions were used in models in the Discussion. Mean points are based on *n*=131 180 tracheids in six stems, and the error bars are the SD.

The probability distribution function (PDF) for the percentage of the tracheid lumen of bin diameter size classes is important for modeling PEG hydraulics in the discussion. Figure 3 shows the PDF of tracheid diameters and the PDF of sap velocities and sap volume flow rate in the tracheids, assuming the velocity is proportional to the diameter squared, and the flow rate is proportional to diameter to the fourth power (Poiseuille’s law).

### Flow rate and concentration (Brix) change with time in the sample

**Fig. 4.**
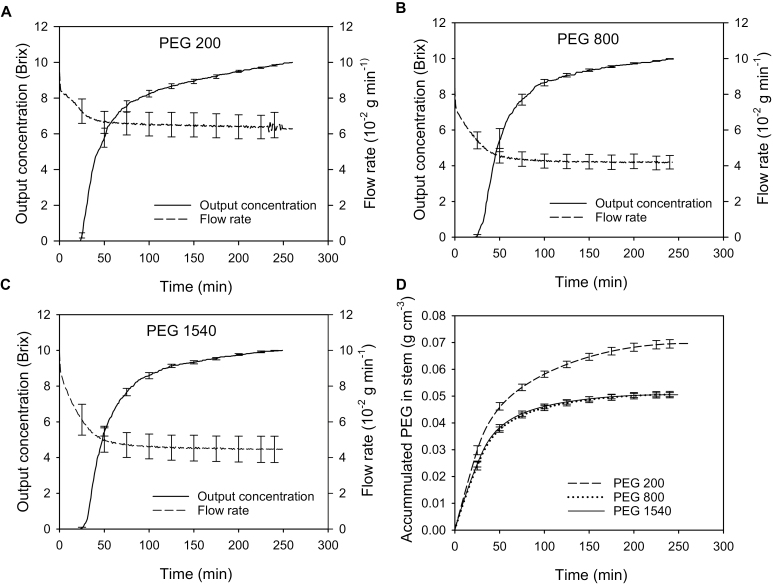
(A–C) The mean flow rates and output concentrations of the different molecular weight PEG solution change with time during the experiment in six *Metasequoia* stem samples. (A) PEG200, (B) PEG800, and (C) PEG1540. (D) The average accumulated PEG mass per cm^3^ of stem in six stems over time in *Metasequoia* stem based on (A–C) and *M*_PEG_/stem volume using Eq. (1). Bars are the SE.

The flow rate of the PEG solution sharply decreased with time, and then slowly changed until it was nearly stable; the flow rate change with time was explained by the replacement of low viscosity water in tracheids with a higher viscosity PEG solution. The output concentration remained zero for 25–27 min, then began to sharply increase with time, followed by a decrease in the slope to zero at equilibrium when the concentration injected equaled the efflux concentration at the stem’s distal end (Fig. 4A–C). The stable flow rate in the PEG200 solution was about 1.5 times that with PEG800 and PEG1540, but the latter two were not significantly different (*P*>0.05). The mean tempo of the output PEG concentration of PEG800 and PEG1540 was not significantly different from that of PEG200 (Fig. 4A–C) except for minor differences for two periods: 27–47 min and 90–120 min.

### The tempo of PEG mass accumulation in the sample

The qualitative trend of PEG mass accumulation with time in the stem was similar among PEG solutions of different MWs. Initially, the PEG accumulation sharply increased because 10 Brix PEG was flowing in and zero was flowing out for the first 25 min; then, the net rate of increase slowed down and gradually became zero (Fig. 4D). The mass of accumulated PEG200 was significantly higher than the high-MW PEG solutions after 30 min. The tempo of accumulated PEG800 versus PEG1540 had no significant difference during the whole experiment (Fig. 4D dotted and solid lines). All the results proved that PEG200 molecules accumulated more in the cell walls in the stem than did PEG800 and 1540.

## Discussion

### Water distribution in stem tissue

This study estimated the water content of lignified cell walls in biologically active wood and the fraction of cell-wall water that might be ‘solute-free space’. Woody tissue always contains a certain volume of living cells (ray, pith, and xylem parenchyma cells). After spinning water out of the tracheid lumina with a centrifuge and after drying the samples for >2 d at 75 °C, the remaining water was gravimetrically determined to be 23.9% of the tissue volume for *Metasequoia*. After the centrifugation process, the pith was shrunken (Fig. 2), and the ray cells were tightly packed with starch (amyloplasts); hence, the amount of water in living tissue was likely to be quite small.

How much water might be in living wood cell lumina (ray cells and parenchyma cells excluding the cell wall) of *Metasequoia*? In our study we selected *Metasequoia* because it had the smallest fraction of living cells in sapwood of all the candidate species we examined. Our centrifugation technique allowed us to strip away water from tracheid lumina leaving only water in cell walls plus a small volume fraction of living cells. There are no techniques to separate water from cell walls and water inside living cells; nevertheless, it is still valuable to estimate how much of the fraction of the water ascribed to cell wall water is in fact in the living cell lumina. We guess that the ray cells contain 25% water inside them because of the volume occupied by starch, and the pith cells decrease to approximately 15% water after spinning because of cell collapse (Fig. 2). Based on these estimates, we set the upper bound of living cells’ water content at 1% of wood volume, leaving a cell-wall water content of 22.9% of the wood volume. Perhaps cleverer experiments can be devised to obtain more precise estimates in the future. Assuming the wall contains 22.9% water, the cell wall is 37.6% water by cell-wall volume compared with the value in Table 2 (*W*_cw_/VF_cw_=39.2%).

### PEG solute distribution kinetics

We first address the kinetics of the PEG200 accumulation because it is accumulated to a higher mass than PEG800 and PEG1540. This result suggests that the smaller PEG molecule is accessible to a greater water volume (solute-free space) than the larger molecules.

Models were evaluated to account for the tempo of PEG solute concentration versus time in experiments such as those in Fig. 4A. More than 131 000 tracheid lumen areas were measured to obtain the mean diameters shown in Table 2 and Fig. 3, so these data could also be used to compute the number of tracheids having lumen diameters in the 1 μm-wide bin size classes ranging from 3.5 to 27.5 μm.

Two models were run to evaluate how the lumen diameter regulated the velocity of PEG solution movement assuming a unit-pipe model. In the unit-pipe model, tracheids of any diameter are connected to like-diameter tracheids for the total length of the stem samples (27.3 cm), and no water movement between diameter size classes was allowed. PEG would emerge from the largest diameter tracheids first and mix with pure water from the smaller tracheids at the stem’s distal end. With time, smaller tracheids distribute PEG to the stem’s distal end and raise the concentration of PEG because of less dilution. In the first model, PEG was transported in the pipes without moving into the cell walls. The experimental flow rates in Fig. 4A were used to partition velocities (cm min^−1^) and flow rates (g min^−1^) in unit pipes using the PDF distributions shown in Fig. 3. The flow velocity determined the time delay until PEG began to emerge from each bin diameter size class; then, the flow rates/volumes for all unit pipes were summed to compute the mixed value of PEG concentration emerging from the stem’s distal surface.

**Fig. 5.**
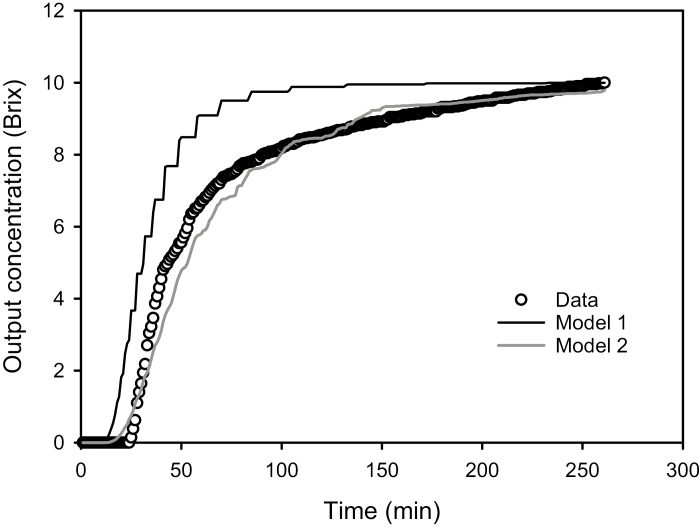
Experimental data and model outputs for diffusion of 200 MW PEG. Model 1: the PEG moves through the tracheid lumina without moving into the cell walls. Model 2: the solutes diffuse into the cell walls as the PEG solution moves in the unit pipes. See text for model details.

The first model fit the data more poorly than the second model, but both models were relatively good at mimicking the approximate shape of the concentration tempo (Fig. 5). The second model differed from the first only in that the unit pipes exchanged PEG by diffusion through water in the adjacent cell walls. The rate of diffusion could be very fast, as explained below.

The average distance by which molecules diffuse in water, *x* (cm), depends on the diffusion coefficient, *D* (cm^2^ s^−1^), and the time, *t* (s), according to *x*^2^=2*Dt*. A tabulation of PEG diffusion coefficients in water is shown in Table 3. The average wall thickness in tracheids is 7×10^−4^ cm, but since PEG diffuses from both adjacent tracheid lumina, we can assign *x*=3.5×10^–4^ cm to compute *t*. The value *D* of O_2_ diffusion in wood has been measured (Sorz and Hietz, 2006) and was two orders of magnitude less than in water. From this, we can conclude that the *D* values of PEG would also be approximately two orders of magnitude less, and this assumption would yield correspondingly longer *t* values of approximately 1.5–4.3 s. The value of *D* for a fluorescent dye (MW 558) made by Canny (1990) in lignin-free walls was lower and was the only value we could find for a solute’s diffusion in primary cell walls. However, all estimated times were short in comparison with the experimental time steps (1 min), so the second model assumed instantaneous mixing by diffusion of PEG between the tracheid lumen and the adjacent wall in each time interval of 1 min. Model 2 resulted in an acceptable agreement with experimental data.

**Table 3.** Diffusion coefficients in water

		In water		In wood		In primary wall	
Solute	MW	*D*	*t* (ms)	*D*	*t* (s)	*D*	*t* (s)
O_2_	32	2.00×10^−5^	3.1	1.00×10^−7^	0.61	2.1×10^−9^*	3
PEG200	200	8.00×10^−6^	7.7	4.0×10^−8^*	1.5	5.0×10^−9^*	12
Dye	558	4.8×10^−6^*	12.8	2.4×10^−8^*	2.6	3.00×10^−9^	20
PEG800	800	4.00×10^−6^	15.3	2.0×10^−8^*	3.1	2.5×10^−9^*	25
PEG1540	1540	2.00×10^−6^	30.6	1.4×10^−8^*	4.3	1.5×10^−9^*	41

O_2_ in water is from the *Handbook of Physics and Chemistry*, the PEG values were measured in water by Shimada *et al.* (2005), and the value for O_2_ in wood is from Sorz and Hietz (2006). The dye (sulforhodamine B, MW 558) was measured by Canny (1990) in wheat leaf primary cell walls. *Extrapolated in each column using *D* ∝ MW^−0.5^ and assuming no MW exclusion from cell walls.

### PEG solute distribution (mass)

The equilibrium PEG solute accumulations were highest for the PEG200 and least for PEG800 and 1540. The latter two were not significantly different (Fig. 4D). The final values were approximately 0.07 g PEG cm^−3^ of wood for PEG200 and 0.05 g PEG cm^−3^ of wood for PEG800 and 1540. The parameters in Table 4 were used to calculate the volume of PEG solution per cm^3^ of wood that would contain the terminal weights of PEG in Fig. 4D. Hence the exclusion of solutes begins somewhere between MW 200 and 800.

**Table 4.** Parameters for calculation the volume of PEG solution per cubic centimeter of wood volume

PEG MW	Final g PEG cm^−3^ wood	Density (g PEG solution cm^−3^)	% PEG ((g PEG/g solution)×100)	Volume fraction of wood volume (%)	VF_tr_+*W*_cw_ (%) (from Table 2)
200	0.0696	1.015	12.28	55.84 ± 1.26	57.61 ± 1.6
800, 1540	0.0505	1.014	11.27	44.19 ± 1.04	
				Difference: 11.65%	

Volume fraction as % wood in column 5 was calculated from the values in column 2–4 from: col. 5=col. 2/(col. 3×col. 4).

This is in striking contrast to a large literature base from the wood technology sciences (see Introduction). In many of these studies (reviewed by Hill and Papadopoulos, 2001) the MW cut-off is much higher. For example, Tarkow *et al.* (1966) used boiling water to extract polymers from Sitka spruce heartwood and found that PEG up to MW 2000 penetrated the resulting boiling-extracted cellulose water, but boiling extracts many polymers in lignified wood (Li *et al.*, 2017), so the penetration of larger molecules can be explained by the removal of lignin and other polymers during the boiling extraction process before PEG is equilibrated. Since the purpose of this study was to speculate on the functional role of pits between living ray cells and dead xylem elements, we think the large literature review based on studies of dead wood tissue (heart-wood) and cellulose extracted to yield pulp provides little basis to speculate on the functional significance of pits in living wood connected to biologically active ray cells and dead tracheids.

The volume of solution containing 10 Brix PEG200 was not significantly different from the anatomically and gravimetrically determined water content in the combined water space of the cell wall and the tracheid lumina (VF_tr_+*W*_cw_, %). However, the higher MW PEG was excluded from 11.65% of the woody tissue space. This fact means that the solute-free space for low-MW PEG is more than that for high-MW PEGs. The total water fraction in the stem’s cell wall was 23.89% of the wood volume (Table 2). We think it is likely that the 11.65% of exclusion zone can be identified as the water in the lignified walls because lignin would reduce the space between cellulose fibers and hence exclude high-MW solutes. The functional importance of pits is easily inferred since there is no direct path for, say, sucrose between ray cells and tracheid lumina except via pits because of the exclusion of large solutes from lignified wood.

### Experimental evidence for semipermeability of lignified cell walls for a solute of MW >300 in angiosperms

The above results confirm that lignified walls have one of the properties of a semipermeable membrane: exclusion of high-MW solutes. A semipermeable membrane excludes water less than solutes, thereby allowing water to pass through faster than solutes. A semipermeable wall displays the property of ‘osmosis’, i.e. water flows into the region with the higher concentration of non-permeant solutes. However, if a pressure equal to *RT*Δ*C* (where *R* is the gas constant and *T* is the absolute temperature) is applied to the high concentration side, the osmotic flow stops. This process happens even in systems without lipid–protein membranes. We must look for wood structures that have lignified walls between cells with no membranes and no primary walls exposed (no pits) for verification.

There is experimental evidence that lignified cell walls are permeable to water but not to high-MW solutes from numerous studies of *Acer* and *Juglans* species. The evidence comes from microscopic examination of the movement of fluorescent dyes in woody species without pits connecting vessels to wood fibers (libriform fibers in certain species). Most species of angiosperm trees have pits interconnecting all major cell types in all combinations between ray cells, vessels, and fiber tracheids. So, any cell type can be connected to any other cell type via pits. However, in *Acer* and *Juglans*, pit connections between libriform fibers and vessels are absent, and this causes osmotically induced pressures between libriform fibers filled with water plus air bubbles and vessels filled with water and sucrose (MW 342) (Tyree and Zimmermann, 2002).

Cirelli *et al.* (2008) performed microscopy studies with strongly fluorescent dyes (fluorescein, MW 376 or 389 depending on the chemical form). When the dyes were injected into *Acer* and *Juglans* stems via vessels, the dyes could be seen in vessels but not in libriform fiber tracheids even though the libriform fiber tracheids had water in their lumina. In contrast, when injected into *Betula* stems, the dyes were seen in the wood fiber tracheid cells and presumably got there via the pits observed between vessels and fiber tracheids.

Many other experiments have demonstrated that six-carbon sugars, mannitol (MW 180), urea (MW 80), and salts (MW <100) can permeate lignified walls between vessels and fiber tracheids but 12-, 18-, and 24-carbon sugars (MW 342–675) all produce a stem ‘turgor’ pressure or osmosis consistent with the semipermeable transport of water versus high-MW sugars across lignified walls (Tyree, 1983; Johnson and Tyree, 1992; Tyree and Yang, 1992; Yang and Tyree, 1992; Tyree and Zimmermann, 2002). This phenomenon cannot be repeated in species with pits between vessels and fiber tracheids, because if there were pits (without lignin), then 12-carbon sugars are likely to permeate. And this tendency of lignin to exclude large molecules is consistent with evidence from studies on porosity of pulp used in paper-making processes in which lignin and other high-MW polymers were removed to allow quite large molecules, up to MW 2000, to permeate (Hill and Papadopoulos, 2001).

The size of a sucrose molecule, including its hydration shell of 12 water molecules, is approximately 0.9–1.3 nm. In contrast, the porosity of primary cell walls (non-lignified) has been estimated at 3–5 nm (Carpita *et al.*, 1979). Thus, we can expect the porosity of lignified walls to be <1 nm to prevent the permeation of sucrose and higher MW solutes, but the permeability of sucrose across primary cell walls can be understood based on Carpita’s results.

There is quite a large literature base in wood science journals related to the pulp and paper industry, which is easily accessible in the reviews of Hill and Papadopoulos (2001) and Shi and Avramidis (2018) and the research papers of Kojiro *et al.* (2010) and Kulasinski and Guyer (2016). Virtually all the research in this review addresses the cell wall properties of dead heartwood and mostly in wood samples extracted as pulp for paper manufacturing. These extraction techniques remove lignin and cause ‘wood fibers’ (tracheids of conifers) to separate from each other to form pulp. Other approaches focus on nano-pore structures deduced from gas adsorption measurements in completely dried wood (Li and Wang, 2013). Our study is focused on living wood, ‘sapwood’, in branches no more than 3-years old. As can be seen here, living wood is likely to have different properties than heartwood (extracted for 2 d in boiling water) and pulp (extracted from heartwood by the Kraft process using strong NaOH and Na_2_S at >170 °C to remove lignin). Nevertheless, some techniques used in the wood technology, pulp, and paper industries are potential candidates for new and innovative approaches that might be adapted to studies of solute transport in living woody tissues.

### Implications of this research for the function of ray-cell pits in solute transport

Wood has functions of water/salt transport from roots to shoots and carbohydrate storage and retrieval between stems and shoots. In combination with the bark, stems provide annual cycling of assimilates. Assimilates are transported in sieve tubes, then unloaded via companion cells to ray cells in the phloem. Ray cells have a continuous symplasmic transport mechanism from the bark to the wood, where starch is stored through the growth season in living cells. Additionally, during the entire growth season, sugars are transported together with a significant concentration of >100 mM of combined salts. The salts must be offloaded from sieve tubes with the sugars, and these salts must be recycled to the leaves via the wood. The sugars are stored as starch in winter and then allocated to growing shoots/leaves in spring. Since xylem sap in spring often contains >30 mM sugar (>1% by weight), it is presumed that it is released into the xylem from ray cells (Marvin and Erickson, 1956; Marvin, 1958). In some genera, such as *Acer* and *Juglans*, the xylem sugar concentrations can reach 100 mM or more.

When sugars and salts are exported from ray cells, the initial flux across the membrane is generally carried by membrane-bound proteins that enhance solute flux, but once the solutes reach the outer surface of the membranes, an apoplastic transport pathway remains via diffusion through cell-wall water.

Our paper enhances understanding of solute transport through cell walls. We know the volume fraction of water in cell walls, and not all the volume fraction is available for transport of high-MW solutes. The question is, can we use Fick’s law for diffusion through cell walls to confirm a functional significance of pits? Do pits enhance the efficiency of solute transfer from the outside surface of the plasma membrane to the flowing water in xylem conduits? The efficiency or functionality can be measured in terms of the Δ*C* needed for diffusion to match the likely membrane efflux rate of solutes, *J*_s_. A functional pit would have a relatively small Δ*C.*

Lignified cell walls are usually thicker than primary cell walls found in pits and impermeable to molecules the size of sucrose or larger. What about the pit cell walls? How efficient are they in the transport of solutes such as sucrose?

We can use Fick’s law of diffusion to address this question:

$$\begin{array}{l} J_{s}=\;D\Delta C/\Delta X \end{array}$$ (2)

where *J*_s_ is the flux rate in mol s^−1^ cm^−2^, Δ*C* is the concentration drop across the cell wall driving the diffusion (mol cm^−3^), and Δ*X* is the thickness of the cell wall (cm). Using Eq. (2), we calculated that the Δ*C* across the pit wall would be ≤6.6 mM by diffusion; the calculation assumed a 1 cm length of ray cells with a 10 µm width, square sides that contained 50% starch by volume, and all starch mobilized through pits in 1 week through pits occupying 5% of the wall surface. The Δ*C* values are sufficiently small to allow for the efficient transfer by diffusion across pit membranes even given high membrane fluxes and small flux areas (see Supplementary Appendix S1 at *JXB* online for the calculation details).

## Conclusions

Authors of plant anatomy books have often assigned a function to pits based on the strategic location of pits between living cells and xylem, but obtaining direct proof has proven challenging. While the function of pits is well understood in dead xylem cells (between conifer tracheids or angiosperm vessels) and is well documented (Tyree and Zimmermann, 2002), the function of pits between ray cells and dead xylem elements is harder to prove. The functional role of pits implies that (i) most solute transport could occur through pits and (ii) permeation of solute through lignified regions is somehow excluded or occurs at a dramatically restricted rate. The purpose of this research was to quantitatively measure the solute-free space of *Metasequoia* stems. The hypothesis was that the volume of solute-free space should decrease as the solute MW increases. Tracheid lumina could hold enormous molecules if they could only get inside. Lignified cell walls could hold and allow permeation of only low-MW molecules because the space between polymer chains in lignified wood is quite small. The combined anatomical and physiological evidence in this paper lends support to the hypothesis.

A review of the literature revealed that the semipermeable cut-off MW in lignified cell walls was below that of sucrose (MW 342) and above that of PEG200 (MW 200), and a literature review has provided additional proof. Some species have no pits connecting vessels to fiber tracheids and that absence of pits results in unusual osmotic properties of the stems. We hope that the preliminary success we have had with our techniques will inspire others to think of even better ways to prove the functional significance of pits in the transport of water and solutes in plants.

## Supplementary data

Supplementary data is available at *JXB* online.

Appendix S1. Computation of Δ*C* across primary pit walls during sugar retrieval from ray cells.

eraa058_suppl_Appendix_S1
