# Supplementary material for: Inferring the role of pit membranes in solute transport from solute exclusion studies in living conifer stems
Source: J Exp Bot. 2020 Feb 5;71(9):2828–37. doi: 10.1093/jxb/eraa058 (PMC7210765; doi:10.1093/jxb/eraa058)
Supplement: eraa058_suppl_Appendix_S1 [file eraa058_suppl_appendix_s1.pdf]

Supplementary Appendix S1.

Computation of ΔC across primary pit walls during sugar retrieval from ray cells

The largest uncertainty in the calculation is approximating the value of *D* for sucrose diffusion in the primary cell wall from the diffusion coefficient of a dye in a wheat-leaf primary cell wall. Canny (1990) measured the rate of diffusion of a fluorescent dye (sulforhodamine B, MW 558) through the primary cell walls of wheat leaves and computed values between 10^{-9} and 6×10^{-11} cm^2 s^{-1}.

We estimate the diffusion coefficient of sucrose in primary cell walls to between 1E-9 and 3E-10 cm^2 s^{-1} from Table 3 and the MW of sucrose. Ray cells are 10 µm square in the lumen area. So, ray cells 1 cm long would have a volume of 1E-6 cm^{-3}. The equation that applies to computing ΔC *is* Δx J/D_s from Fick's law, where Δx is the cell-wall thickness and D_s is the coefficient of sucrose diffusion in the cell wall. We also estimate $J = M/(A_p\ \Delta t)$, where *M* is the mass of sucrose to be exported over across pit area, A_p, in time period Δt, which is usually a few weeks (6.05E5 s per week).

$$\Delta C = \frac{\Delta x}{D_s}\frac{M}{A_p \Delta t} \qquad \text{Eq. (S1)}$$

In the estimate below, we use an upper bound value for Δx and lower bound estimates for A_p and Δt.

If half that volume was starch at a density of 1.54 g cm^{-3}, it would contain 7.7 E-7 g of starch. A mass of 324 g of starch yields 1 mole of sucrose, so the cell contains *M* = 2.38E-9 moles of sucrose. Normally the ray cells convert starch to sugar over a one-month period in spring to fuel leaf growth, but we can apply 1 week = 6.05E5 s in the flux calculation. If the pits occupy just 5% of the ray-cell surface area in ray cells 1 cm long and 10^{-3} cm wide square, then the flux would occur across A_p = 2E-4 cm^2 because sucrose cannot permeate lignified walls (see the previous section). So the flux would be = 2.38E-9 mol/(6.05E5 s*2E-4 cm^2) = 1.96E-11 mol s^{-1} cm^{-2}. D_s, which is different for different solutes and proportional to the $MW^{-0.5}$, and when reduced by a factor of 10 for good measure, is likely to be 3E-10 cm^2 s^{-1}. Solving Eq. (2) for ΔC across the cell wall of 10^{-4} cm Δx, we obtain approximately 6.6 mM. If the conversion rate of starch

to sugar in ray cells in spring requires up to the full 6 weeks of rapid leaf growth, then the ΔC could be 6 times less. Many primary walls in the pit are thinner than the value used in this calculation. Using a smaller value would proportionally reduce ΔC. Any consideration that reduces the J_s value also reduces ΔC; these include an extraction period longer than 1 week to match the 4 to 6 weeks for rapid leaf growth in spring or an increase in the percentage of area occupied by pits in ray cells beyond the conservative 5% in the above calculations.

The above calculations are quite speculative and hence do not deserve much more elaboration. For example, almost nothing is known about salt transport into and out of ray cell. But ray cell function is tied to carbohydrate storage and retrieval. Storage occurs during the growth season, and retrieval happens during the early spring to fuel early growth of leaves and wood. The sucrose concentration drop (6.6 mM) occurs across the pit membranes (primary cell walls) and the concentration drop is generated by the active transport of sucrose across the membranes on the inner surface of the pit membranes.
